# Impact of RTS,S/AS02_A_ and RTS,S/AS01_B_ on Genotypes of *P. falciparum* in Adults Participating in a Malaria Vaccine Clinical Trial

**DOI:** 10.1371/journal.pone.0007849

**Published:** 2009-11-17

**Authors:** John N. Waitumbi, Samuel B. Anyona, Carol W. Hunja, Carolyne M. Kifude, Mark E. Polhemus, Douglas S. Walsh, Chris F. Ockenhouse, D. Gray Heppner, Amanda Leach, Marc Lievens, W. Ripley Ballou, Joe D. Cohen, Colin J. Sutherland

**Affiliations:** 1 Walter Reed Project, Kenya Medical Research Institute, Kisumu, Kenya; 2 Division of Malaria Vaccine Development, Walter Reed Army Institute of Research (WRAIR), Silver Spring, Maryland, United States of America; 3 GlaxoSmithKline Biologicals, Rixensart, Belgium; 4 London School of Hygiene and Tropical Medicine, London, United Kingdom; Walter and Eliza Hall Institute of Medical Research, Australia

## Abstract

**Objective:**

RTS,S, a candidate vaccine for malaria, is a recombinant protein expressed in yeast containing part of the circumsporozoite protein (**CSP**) sequence of 3D7 strain of *Plasmodium falciparum* linked to the hepatitis B surface antigen in a hybrid protein. The RTS,S antigen is formulated with GSK Biologicals' proprietary Adjuvant Systems AS02_A_ or AS01_B_. A recent trial of the RTS,S/AS02_A_ and RTS,S/AS01_B_ vaccines evaluated safety, immunogenicity and impact on the development of parasitemia of the two formulations. Parasite isolates from this study were used to determine the molecular impact of RTS,S/AS02_A_ and RTS,S/AS01_B_ on the multiplicity of infection (MOI) and the *csp* allelic characteristics of subsequent parasitemias.

**Design:**

The distribution of *csp* sequences and the MOI of the infecting strains were examined at baseline and in break-through infections from vaccinated individuals and from those receiving a non-malarial vaccine.

**Setting:**

The study was conducted in Kombewa District, western Kenya.

**Participants:**

Semi-immune adults from the three study arms provided isolates at baseline and during break-through infections.

**Outcome:**

Parasite isolates used for determining MOI and divergence of *csp* T cell–epitopes were 191 at baseline and 87 from break-through infections.

**Results:**

Grouping recipients of RTS,S/AS01_A_ and RTS,S/AS02_B_ together, vaccine recipients identified as parasite-positive by microscopy contained significantly fewer parasite genotypes than recipients of the rabies vaccine comparator (median in pooled RTS,S groups: 3 versus 4 in controls, *P = 0.0313*). When analyzed separately, parasitaemic individuals in the RTS,S/AS01_B_ group, but not the RTS,S/AS02_A_ group, were found to have significantly fewer genotypes than the comparator group. Two individual amino acids found in the vaccine construct (Q339 in Th2R and D371 in Th3R) were observed to differ in incidence between vaccine and comparator groups but in different directions; parasites harboring Q339 were less common among pooled RTS,S/AS vaccine recipients than among recipients of rabies vaccine, whereas parasites with D371 were more common among the RTS,S/AS groups.

**Conclusions:**

It is concluded that both RTS,S/AS vaccines reduce multiplicity of infection. Our results do not support the hypothesis that RTS,S/AS vaccines elicit preferential effects against *pfcsp* alleles with sequence similarity to the 3D7 *pfcsp* sequence employed in the vaccine construct.

## Introduction

The morbidity and mortality attributable to malaria in Africa is reported to be on the decline, thanks to the increased funding from philanthropy and governments [1]–[3] that has enabled deployment of tools such as insecticide-treated nets [4] and artemisinin-based combination therapies [5]. To sustain this momentum and thereby allow African countries to reap the economic benefits that would result from reducing the annual toll of 300 to 500 million malaria cases, a larger armamentarium of malaria control measures are required. There is hope that a malaria vaccine will soon be added to the malaria control toolkit [6].

The most successful malaria vaccine to date is RTS,S, a recombinant hybrid molecule expressed in yeast, in which the partial sequence of circumsporozoite protein (CSP), central tandem repeat, and carboxyl-terminal regions are fused to the N terminal of the S antigen of hepatitis B virus (HBs Ag) in a particle that also includes the un-fused S antigen. These antigens are administered with the AS02_A_, an oil-in-water based Adjuvant System containing the immunostimulants monophosphoryl lipid A (MPL) and *Quillaja saponaria* fraction 21 (QS21; Antigenics, New York, NY, USA) or a liposome based adjuvant system (AS01_B_) containing the same immunostimulants [7]. In experimentally infected volunteers, RTS,S/AS impact on *P. falciparum* parasitemia was evident as either sterile protection or delay in the onset of parasitemia [8]–[13].

In parallel, GSK Biologicals has co-developed with the Walter Reed Army Institute of Research (WRAIR) a more immunogenic formulation of RTS,S based upon the AS01_B_ Adjuvant System. In pre-clinical comparisons to RTS,S/AS02_A_ in mice and monkeys, RTS,S/AS01_B_ elicited equivalent CSP-specific antibody and greater and more sustained cellular immune responses [14]–[16]. Encouraged by these results, we have undertaken comparative trials of RTS,S/AS01_A_ and of RTS,S/AS02_B_ in malaria naïve [13] and malaria experienced adults [17]. In a number of clinical trials, three doses of RTS,S/AS02_A_, RTS,S/AS02_D_ or RTS,S/AS01_E_ have provided children under 5 with a vaccine efficacy of up to 59% against clinical malaria and up to 65.9% against *P. falciparum* infection, [18]–[23].

The CSP is the predominant protein found on the surface of the sporozoite. Studies of the genetic diversity of the gene encoding the *csp* of *P. falciparum* have demonstrated the existence of high levels of genetic polymorphisms in isolates from different areas in Africa [24]. Because of this and the fact that the RTS,S vaccine contains only the *csp* allele of laboratory clone 3D7, it is necessary to determine whether the monovalent 3D7 RTS,S/AS vaccine will elicit a preferential effect against homologous alleles. Such an effect could lead to the development of vaccine insensitive parasite populations and ultimately to the failure of RTS,S-based vaccines [8].

The key polymorphic sites in the *csp* gene which are encompassed by the RTS,S antigen are the T-cell epitopes at the carboxy-terminus of the protein, designated Th2R and Th3R. Polymorphisms in these epitopes were the focus of the evaluation of strain-specificity of RTS,S among adult Gambian men [25]. No strain-specific effect of RTS,S/AS02A on malaria parasites was found, nor were the average number of genotypes (multiplicity) carried during post-vaccination parasitaemia different among vaccinated men and controls. The recent analysis of *csp* sequences and clone multiplicity in 521 parasite isolates from Mozambique children under 5 years who participated in a large Phase II study of the efficacy of RTS,S/AS02_A_ confirmed the previous finding of no significant effect of *csp* sequences but found a small but significant decrease in the multiplicity of infections in post-vaccination parasitemias among those with asymptomatic infections who had received RTS,S/AS02_A_ [18], [26].

The present report is based upon specimens obtained in the context of a comparative safety and immunogenicity trial of two RTS,S formulations, RTS,S/AS02_A_ and RTS,S/AS01_B_, conducted in 255 malaria-experienced adults in Kenya [17]. This original trial examined efficacy as a secondary endpoint, but was not powered to compare clinical efficacy between formulations. In that study, anti-CS antibody geometric mean titers were significantly greater with RTS,S/AS01_B_ compared to RTS,S/AS02_A_, and vaccine efficacy in the RTS,S/AS01_B_ group was 29.5% (95% CI: −15.4 to 56.9) and 31.7% (95% CI: −11.6 to 58.2) in the RTS,S/AS02_A_ [17]. Here, we test for evidence of genotypic selection on the *pfcsp* locus, and for any effect of the RTS,S vaccine formulations on parasite multiplicity.

## Methods

### Ethics statement

Details of scientific and ethical protocol approval for these studies have been reported [17]. The clinical trials gov identifier for this study is NCT00197054 and the GSK Study ID number is 104743 (Malaria-044).

### Study area, population and vaccine trial

The population is primarily Luo who engage in subsistence farming and related industries. Malaria epidemiology in this region is holoendemic. Intense transmission occurs primarily by bites of the *Anopheles gambiae* mosquito. *P. falciparum* parasitemia is present in over 90% of malaria cases. The “long rainy season” of late March through May produces intense transmission from April through August while the “short rainy season” of October through December produces less intense transmission from November through January. Cumulative malaria attack rates are about 95% during the long rains and 75% during the short rains. All individuals in this population are parasitemic multiple times over a lifetime.

**Figure 1:**
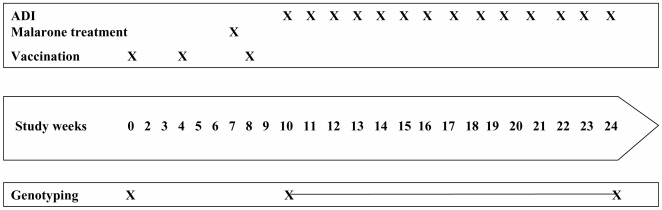
Study events for RTS,S clinical trial in semi-immune adults in Kombewa, Nyanza Province, Western Kenya. ADI = active detection of infection. The ADI period was 14 weeks and began 2 weeks after the third immunization.

Samples for molecular analysis were obtained during the course of a vaccine trial that was carried out in Kombewa district, Nyanza Province, Western Kenya. Witnessed, informed consent was obtained from all adult subjects before screening. Following screening, 255 adult Kenyan volunteers between 18 and 35 years were enrolled for a double-blind randomised controlled trial. 85 volunteers were randomized to each of three groups receiving RTS,S/AS02_A_, RTS,S/AS01_B_ or rabies vaccine (*Rabipur*®; Chiron Behring GmbH) in 3 doses. Enrolment took place in July and August 2005, as the annual “long rains” were ending. Vaccination occurred from August to October 2005, and administration of all three doses to all participants was completed before the “small rains” commenced. One week prior to vaccine Dose 3, all volunteers were presumptively treated with one dose of *Malarone*® (atovaquone and proguanil hydrochloride, GSK, Uxbridge, UK) administered daily for three days under direct observation by the study staff. Blood samples for genotyping were collected at enrollment and during weekly blood draws for active detection of infection (ADI) that started two weeks after dose 3 of *Malarone* and continued for 14 weeks (Figure 1). Samples were also collected during passive case detection in all volunteers presenting with symptoms consistent with malaria during the same observation period.

### Blood sample collection and DNA extraction

Whole blood collected in K_2_ EDTA tubes were labeled with unique sample identifier number. DNA was extracted from the K_2_ EDTA blood using QIAamp DNA Blood mini Kits (QIAGEN Inc., Valencia, CA, USA). DNA samples were stored at −80°C until use.

### CSP genotyping

Primers that bind to conserved sequences flanking the Th2R and Th3R region of *P. falciparum* csp gene were used in a nested PCR to amplify a 361 base pair fragment covering nucleotides 826 to 1186 of the csp gene (numbering from the first ATG of the 3D7 pfcsp mRNA sequence PFC0210c accessed at www.plasmodb.org) using previously described primers and conditions [26], as follows. The PCR reagents were obtained from Applied Biosystems (Banchburg, NJ, USA). In the first-round of PCR, amplification was carried out in a 20 µL reaction volume containing 1× PCR buffer, 1.5 mM MgCl_2_, 250 µM of each dNTPs, 2 µL of genomic DNA extracted from 200 µL of whole blood, and 1 U of AmpliTaq Gold. Primers were used at a concentration of 300 nM each. Amplification was performed in a DNA Thermal Cycler (Tetrad PTC-225, MJ Research Inc., Watertown, Massachusetts, USA). The amplification conditions were: an initial denaturation of 95°C for 3 minutes followed by 40 cycles of 1 minute at 95°C, 1 minute at 60°C and 1 minute at 72°C followed by a final extension step at 72°C for 10 minutes. The second-round of PCR contained 1 µL of the primary amplicons in 200 nM of each primer in 1× buffer as above. Amplification conditions were as for the primary PCR. 5 µl of the 2^nd^ round PCR products were monitored on a 2.0% agarose gel in 1xTAE buffer to check the quality, size and yield of the PCR products before proceeding to product purification and sequencing, using between 10 and 120 ng of PCR product as previously described (26). The sequence of every PCR product was proof-read on two strands by the same investigator. A second investigator read a random QC sample of ∼40% of all sequences on both strands, and also checked all sequences deemed to be mixed or ambiguous independently from raw sequencer output. For each double-read sequence a consensus was reached or the assay repeated. Data was collated from all isolates with a single *csp* allele, or where a clear majority allele could be unambiguously identified. In mixed genotype infection, a majority clone was recognised if, at each mixed allele polymorphic nucleotide position, one residue dominated over others present, and this dominance was clearly discernable on both DNA strands.

### Genotyping of msp-1 and msp-2 for determination of MOI

Allelic family-specific, nested PCR of 2 polymorphic regions of *P. falciparum* genes, namely *msp*-1 block 2 and *msp*-2 block 3 were used to detect the genetic structure of the parasite populations essentially as previously described [27] with minor modifications of primer annealing temperatures to optimize performance in our PCR machine (annealing temperatures for nest 1 amplification were 56°C for *msp* -1, and 58°C for *msp*-2; nest 2 annealing temperatures were 60°C for *msp*-1 and 65°C for *msp*-2 ). Following electrophoresis, staining with ethidium bromide and observation under ultra-violet illumination, bands corresponding to different parasite allelic forms were distinguished and counted and the number of genotypes for *msp*-1 and *msp*-2 loci determined. Gels were double-scored independently by 2 investigators, and either consensus reached or the assay repeated. Each band seen in a gel lane was considered to represent a *P. falciparum* population that shares a single allelic variant, i.e. this population represents a distinct genotype. The minimum number of genotypes at each locus was determined for each sample and the results entered in an excel worksheet.

### Data processing and statistical analyses

Analyses were performed according to a plan developed prior to data collection. Data were analyzed for three cohorts in an according to protocol (ATP) analysis: RTS,S/AS02_A_ recipients, RTS,S/AS01_B_ recipients, and rabies control vaccine recipients. We also analysed pooled data from both RTS,S/AS02_A_ and RTS,S/AS01_B_ recipients. The endpoint for assessing strain specificity of the vaccine was the relative proportion of non-vaccine type (3D7/NF54) alleles for each of the polymorphic amino acids sites at Th2R and Th3R in the combined RTS,S group versus controls. The primary analysis included single and clear majority alleles and the combined RTS,S group was compared to controls by Fisher exact test. Secondary analysis included comparisons of each of the vaccine formulations (RTS,S/AS01_A_ and RTS,S/AS02_B_) versus control as well as an analysis taking into account mixed alleles by including all available sequence data (data not shown; analysis did not alter results as there were few such infections in our dataset).

The distribution of the number of amino acids in the Th2R and Th3R region different from vaccine type was also calculated and groups were compared by Wilcoxon Rank Sum tests. Descriptive statistics of the number of genotypes in each allelic family of *msp*-1 and *msp*-2 (MOI) were computed and groups were compared by Wilcoxon Rank Sum tests and Gamma regression analysis adjusted for parasite density and age (as previously described) [26]. Primary analysis compared pooled RTS,S groups versus control group, while secondary analyses compared each vaccine formulation versus controls. All tests are 2-sided at 5% significance level and no adjustments for multiplicity were applied. Analyses were performed in SAS, version 9.1.

## Results

### Study events and timing

Study events are summarized in Figure 1.

### Samples analyzed

The study enrolled 255 subjects that were randomized into three study arms of 85: RTS,S/AS01_A_, RTS,S/AS02_B_ and rabies vaccine. At enrolment, 44 scheduled samples were not evaluable either because of PCR failure, had clotted or were not collected. Of the remaining 211 samples, 191 individuals (90.5%) were found to harbor *msp1* and/or *msp2* alleles as detected by PCR and their distribution in the vaccine cohorts is shown in Table 1. Single or clear majority *pfcsp* allele sequence was obtained from the *csp* gene of 146 of 211 (69.2%) of these pre-immunization specimens. During ADI and at cross-sectional surveys, 93 individuals were identified as having malaria parasites as detected by blood film. Eighty-five of these (91.4%) were successfully evaluated for MOI and 87 (93.6%) for *csp* sequences and their distribution in the vaccine cohorts is shown in Table 1.

**Table 1:** Summary of sample numbers used for multiplicity of infection and csp sequence analysis.

Sample numbers	RTS,S/AS01_B_	RTS,S/AS02_A_	Rabies vaccine	Total
Enrolment MOI	65	65	61	191
Enrolment *csp*	49	49	48	146
ADI MOI	22	26	37	85
ADI *csp*	22	28	37	87

### Multiplicity of infection

**Figure 2:**
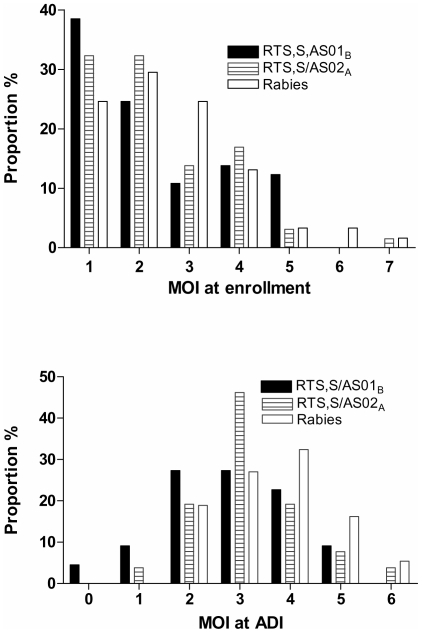
Multiplicity of infections at enrollment and during active detection of infection. Minimum number of clones for vaccine and control groups at enrollment and during the active detection of infection (ADI) period. At enrollment, denominators were n = 65 for RTS,S/AS01_B_, 65 for RTS,S/AS02_A_ and 61 for rabies. During the ADI period, the denominators were n = 22 for RTS,S/AS01_B_, n = 26 for RTS,S/AS02_A_ and n = 37 for rabies.

The number of distinguishable alleles for MSP1 and MSP2 genes was determined for each parasite isolate and the largest of these numbers was considered the “multiplicity of infection” (MOI) of that sample. Figure 2 shows the distribution of MOI at enrollment (panel A) and during ADI (panel B). At enrollment, the mean (Standard Deviation) MOI was similar in all groups (RTS,S/AS02_A_: 2.32 (1.31), RTS,S/AS01_B_: 2.37 (1.43), and rabies vaccine 2.57 (1.40); median 2 for all groups). Incident infections identified by blood film during post-vaccination follow-up displayed a higher overall MOI than was observed at enrollment (Figure 2, panel B). Also, during the ADI period, a significant reduction in MOI was observed in the pooled RTS,S (median 3) versus controls (median 4) (p = 0.031). Modeling MOI by Gamma regression models confirmed these results (unadjusted p = 0.038, adjusted p = 0.019 for parasite density and age). The reduction in MOI was more pronounced for the RTS,S/AS01B group (p = 0.034) than for the RTS,S/AS02A group that did not reach statistical significance (p = 0.130).

### Th2R and Th3R sequences

At enrolment, isolates were genotyped at the 2 polymorphic regions of the *csp* gene to determine the prevalence of 3D7-like alleles compared to that of non-3D7-like alleles at each polymorphic amino acid position in the Th2R and Th3R epitopes in the vaccine and control groups.

**Figure 3:**
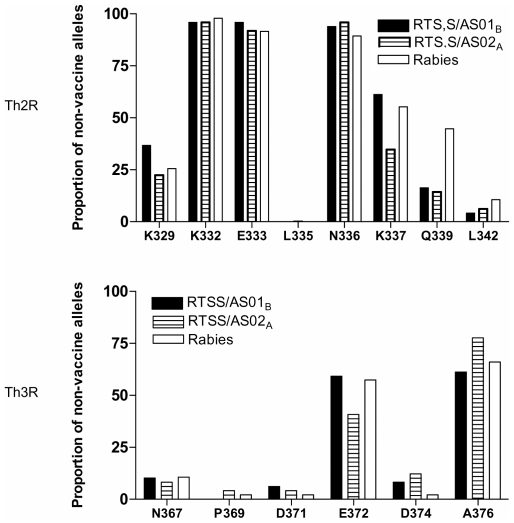
Distribution of *csp* non-vaccine type alleles at enrollment. Percentage distribution of the polymorphic amino acids sites in the Th2R and Th3R regions in the RTS,S vaccine and control groups at enrollment.

Figure 3 shows the proportion of isolates that contained non-vaccine residues in the *csp* Th2R and Th3R at enrollment. Overall, the prevalence of non-3D7 alleles at particular amino acids in the 2 polymorphic regions Th2R and Th3R was similar between treatment arms, except for Q339 in Th2R which was at a lower prevalence in the vaccine groups (pooled RTS,S versus control *P = 0.0004*).

Parasites from break-through infections occurring over a 14 week period starting 2 weeks post dose 3 (the ADI period) were also genotyped at Th2R and Th3R *csp* gene to evaluate if the protection conferred by RTS,S is strain specific, i.e. only against parasites with a *csp* sequence similar to vaccine type (3D7). No *pfcsp* allele identical to the 3D7 allele used in the vaccine construct was found in any of the break-through infections (during ADI) in the three arms of the study (Supplementary Figure S1). The average number of differences to the vaccine allele was 8 (range = 6–12) and was similar between treatment groups (pooled RTS,S versus control *P = 0.904*).

**Figure 4:**
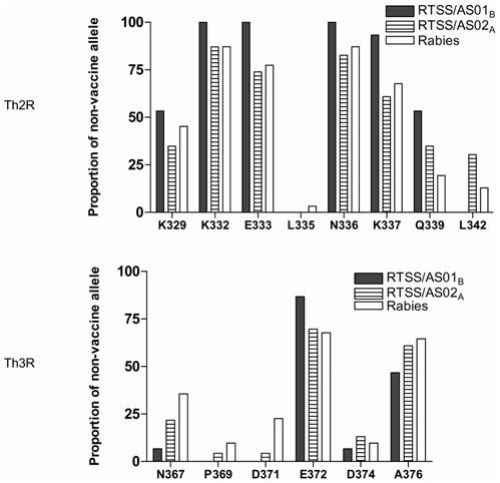
Distribution of *csp* non-vaccine type alleles during period of active detection of infection. Percentage non-vaccine type alleles for the polymorphic amino acids sites in the Th2R and Th3R regions of the RTS,S vaccine and control groups during the ADI period.

Figure 4 shows the proportion of isolates that contained non-vaccine type residues in the *csp* Th2R and Th3R during the ADI period. In the RTS,S/AS01_B_, we observed a significant difference in the prevalence of non 3D7-type Q339 residue in Th2R, which was higher in RTS,S/AS01_B_ recipients as compared to controls (*P = 0.04*). The D371 non-vaccine type residue in Th3R, was less prevalent in the RTS,S groups, this difference reaching statistical significance for pooled RTS,S groups versus controls (*P = 0.02*). There were no other significant differences between the groups at any position in either Th2R and Th3R (Supplementary Table S1 and S2).

### Analysis of pfcsp haplotypes at baseline

We further explored this diversity by examining the prevalent haplotypes at both the Th2R and Th3R sequences among the baseline sample set collected in July 2005, prior to vaccination. A sequence of 8 digits for Th2R and 6 digits for Th3R defined the haplotypes, with 1 indicating vaccine type residue and 0 non-vaccine type residue at each polymorphic amino acid position (Supplementary Table S3). The non-vaccine haplotypes at baseline differed from the vaccine haplotypes at more than one amino acid residual, as previously observed [25]. Among 146 isolates contributing a single or clear majority *pfcsp* sequence, we observed 14 different Th2R haplotypes with 4 common haplotypes accounting for 76.6% of the sequences determined. There were 12 Th3R haplotypes identified, but most of these were rare, with 3 common haplotypes accounting for 82.8% of the sequences. One isolate collected at baseline encoded both Th2R and Th3R epitopes identical to the vaccine sequence.

Table 2 depicts associations among these common haplotypes, showing that the Th2R and Th3R epitope sequences do not randomly assort, and that the possible combinations that can exist in a single *pfcsp* molecule appear to be constrained. In particular there is a statistically significant association between the most common Th2R haplotype (10010111; see Table 2 for explanation of the binary code) and the most common Th3R haplotpye (111110), which indicates these two occur together more often than would be expected if there was random assortment between the two epitope sequences (O.R. 720, 95% C.I. 84.2–6155.1, *P<0.0001*).

**Table 2:** Associations among the most common Th2R and Th3R haplotypes at enrolment, prior to clearance of parasitaemia.

	3 most common Th3R sequences haplotypes		
4 most common Th2R haplotypes	111110 (N = 54)	111011 (N = 37)	111010 (N = 29)
10010111 (N = 49)	48	0	0
10010011 (N = 25)	0	2	20*
00010011 (N = 22)	0	21	0
10010001 (N = 15)	0	0	9

Haplotypes are depicted in binary code across either 8 (Th2R) or 6 (Th3R) variable amino acid positions, where 1 indicates the presence of the 3D7 (vaccine – like) residue, and 0 depicts its substitution with another amino acid. The remaining 45 isolates were comprised of less common Th2R and Th3R sequences.

*16 of the sequences in this category also had the N313K mutation, upstream of the Th2R region.

Interestingly, we also observed 16 sequences with the N313K amino acid substitution upstream of the Th2R epitope, but this mutation did not occur with either of the most common Th2R and Th3R sequences. Rather, all were of a single allele: 10010011 at Th2R and 111010 at Th3R. This identical allele was also common among isolates identified by active detection of infection: of 87 single or majority *pfcsp* sequences determined during post-vaccination, 12 carried the N313K, all of which were 10010011 at Th2R and 111010 at Th3R. However, the most common Th2R haplotype among the baseline samples (10010111) was absent among the 87 ADI isolates. Similarly the Th3R haplotype 111110, which was very common among baseline isolates (Table 2), was found in only 5 of the 87 ADI isolates. These differences suggest that substantial fluctuations in the circulating parasite genotypes can occur between transmission seasons, as previously observed for markers of drug resistance [28]. The distributions among randomized treatment groups of the four most common Th2R haplotypes, and the three most common Th3R haplotypes were not found to differ significantly at baseline from the expected ratio of 1∶1∶1 (*P>0.1* in each case).

## Discussion

In a large clinical trial involving children under 5 years, three doses of RTS,S/AS02_A_ have shown efficacy in extending time to first episode of clinical malaria or *P. falciparum* infection, with measurable benefit still evident 18 months and 48 months later [18], [19], [21]. In further studies of RTS,S/AS02_D_ and RTS,S/AS01_E_ in infants and children up to 17 months of age in Mozambique and Tanzania, efficacy of up to 59% against clinical malaria and up to 65.9% against *P.* falciparum infection was observed [20], [22], [23]. While there are many approaches that can be utilized to improve on this level of protection, for example by combining different antigens that attack the parasite at different life cycle stages, improved adjuvant activity may also provide an increase in immune responsiveness [29]. Recent preclinical trials showed augmented antibody and cell-mediated immune responses when RTS,S was combined with a new Adjuvant System (RTS,S/AS01_B_) compared to another formulation (RTS,S/AS02_A_) [14]–[16]. Clinical trials that have just been concluded by our institutions have confirmed these findings [13], [17]. In this study, we have analyzed the molecular impact of RTS,S/AS02_A_ and RTS,S/AS01_B_ on MOIs and *csp* T-cell epitopes at baseline (191 isolates for MOI and 146 for *csp*) and during break-through infections (85 isolates for MOI and 87 for *csp*) from a phase IIb trial in Kenyan semi-immune adults conducted in 2005 by Polhemus and colleagues [17].

We used size polymorphisms in the *msp*-1 and *msp*-2 genes to determine the MOI [27]. At enrollment, mean MOI was 2, and was not different between the vaccine or control cohorts (Figure 2) suggesting that at baseline there was a comparable malaria-immune status and that the individuals in these cohorts had recently experienced similar malaria transmission conditions. As shown in Figure 1, all volunteers were presumptively treated with *Malarone*® (atovaquone and proguanil hydrochloride, GSK, Uxbridge, UK) one week prior to administration of vaccine Dose 3 [23] and the presence of malaria parasites were monitored weekly by microscopy from 14 days post dose 3 onwards. All confirmed infections were then evaluated for MOI and for distribution of the amino acid alleles in the Th2R and Th3R region of CSP. Despite the presumptive clearance of malaria parasites, the overall MOI for this period was higher than at enrollment (Figures 2). This may represent differences in parasite detection thresholds, as parasite positive individuals at enrollment were identified by PCR amplification, and thus harboured lower density parasitaemia on average versus positives identified by blood film examination during ADI. Alternatively, seasonal differences in malaria transmission may have contributed to the observed difference in MOI in the two datasets. Break-through infections in RTS,S/AS vaccinees contained fewer genotypes than controls (median 3 versus 4 *P = 0.0313*), a finding that was slightly more pronounced and reaching statistical significance for the RTS,S/AS01_B_ formulation (*P = 0.0340*). However, the difference in the mean MOI between the RTS,S/AS02_A_ and the control was not statistically significant. Overall, our pooled data from both malaria vaccine groups are consistent with other studies that reported reduction in MOI after vaccination with RTS,S/AS02_A_ in Mozambican children [26] but not with the results arising from the trial in Gambian men where a reduction in MOI was not observed.

During ADI, the proportion of isolates with non-vaccine type residues was significantly different in vaccine vs control groups for two individual amino acids: Q339 in Th2R and D371 in Th3R. Non-vaccine type Q339 residue was increased while non-vaccine type D371 was reduced in the vaccine arms. However, since these differences were in opposite directions (Figure 4), and due to multiplicity, it cannot be ruled out that these alterations occurred by chance and therefore do not represent an overall effect. Moreover, analysis of the baseline sequences revealed that, due to chance, differences in prevalence of non-vaccine type residues between treatment groups do occur prior to vaccination as well (Q339). When including mixed alleles, Enosse *et al.* found more isolates with non-vaccine residues at E333-Glu (Th2R) and K337-Lys (Th2R) among the vaccine group compared to controls but conversely found more non-vaccine residues at D374-Asp (Th3R) in the control group [26]. Taking these observations together with our findings at amino acid positions Q339 and D371, we do not find strong support for the hypothesis that either RTS,S formulation significantly alters *pfcsp* gene frequencies, either by selection for escape mutants or deletion of specific vaccine-like genotype subsets. In our Kisumu population, CSP sequences with significant identity to the 3D7 vaccine allele were rare (Supplementary Table S3); the detection of vaccine-mediated shifts in allele frequencies may be more likely in other settings where vaccine-like alleles are more abundant.

It was noted that the most abundant haplotypes in the baseline sample were not common among post-vaccination isolates. Further, certain specific substitutions were more common prior to vaccination, and this was particularly noteworthy for the Q339 non-vaccine allele, which was common in both groups of volunteers that were to receive the malaria vaccine, but by chance was significantly less common among ADI parasite isolates from recipients of the malaria vaccine (fig. 3). However, circulating parasites in all participants were cleared with atovaquone-proguanil treatment prior to the third vaccine dose, and therefore the enrolment parasite population itself was not placed under selection by vaccine-elicited immunity. This discontinuity between pre-vaccination and ADI parasite samples is further exacerbated by the fact that the two samples represent parasites from two distinct, consecutive malaria seasons.

Our descriptive analysis of *pfcsp* haplotypes circulating prior to vaccination provides evidence that the Th2R and Th3R display non-random associations in the parasite population studied. Lack of evidence of random assortment between two loci may indicate functional constraints on the CSP protein that limit the possible amino acid combinations that are viable. It may also simply reflect the close physical association of Th2R and Th3R sequences, such that recombination events between the two sites are extremely rare. Lack of recombination means that novel combinations that arise in either epitope may be successfully propagated, carrying the other epitope along with it unchanged, and so linkage patterns can simply reflect recent evolutionary history. One interesting finding was of a stable *pfcsp* allele - N313K, 10010011 at Th2R and 111010 at Th3R – that was relatively common both in 2005 (baseline sample) and 2006 (ADI sample). This suggests that future studies of anti-CSP vaccines should consider haplotype-based analysis for the evaluation of vaccine-elicited selection [29], [30]. Such analysis requires large sequence sample sets, and was not performed in the current study. It was noted that the most abundant haplotypes in the baseline sample were not common among post-vaccination isolates. We think this difference is best explained by the fact that the baseline sample and the ADI sample represent parasites circulating in two consecutive malaria seasons and thus represents inter-season fluctuations in allele prevalences, as has been observed for drug-resistance-associated loci [28]. All subjects were enrolled prior to the start of the malaria season and therefore infections in the baseline samples most likely represent residual asymptomatic infections resulting from exposure in the previous transmission season.

In conclusion, our results support previous indications that RTS,S/AS vaccines can reduce MOI. This reduction was statistically significant for RTS,S/AS01_B_. However, we are cognizant that attempts to correlate MOI and risk of malaria have been inconsistent [31]–[34] and therefore suggest caution when interpreting any associations between vaccination-elicited protection and effects of adjuvanted RTS,S on subsequent MOI. The sequence data on the Th2R and Th3R region of *csp* presented here do not support the hypothesis that RTS,S elicits strain-specific vaccine selection effects. In future, haplotype-level analyses of larger sample sets as RTS,S vaccines enter Phase III testing may help to establish whether any observed individual amino acid changes in breakthrough parasites represent the beginning of vaccine-dependent cumulative selection for escape mutants of malaria parasites.

## Supporting Information

Figure S1 Clustalalignment of CSP amino acid haplotypes.
(0.22 MB PDF)

Table S1 CSP 3D7/non 3D7 type by polymorphic amino acid site in Th2r (single+majority) (ATP cohort for efficacy)
(0.02 MB PDF)

Table S2 CSP 3D7/non 3D7 type by polymorphic amino acid site in Th3r (single+majority) (ATP cohort for efficacy)
(0.02 MB PDF)

Table S3 Th2R and Th3R haplotype prevalence at baseline and among ADI samples.
(0.08 MB PDF)

## References

[1] A single agenda needed for malaria control (2003). Lancet Infect Dis.

[2] The Global Fund to Fight AIDS Tuberculosis, and Malaria (2006). Funding the Global Fight Against HIV/AIDS, Tuberculosis, and Malaria.

[3] Current Grant Commitments and Disbursements of the Global Fund to Fight AIDS, Tuberculosis, and Malaria. The Global Fund to Fight AIDS Web site.. http://www.theglobalfund.org/en/funds_raised/commitments.

[4] Lengeler C (2004). Insecticide-treated bed nets and curtains for preventing malaria.. Cochrane Database Syst Rev.

[5] Ashley EA, White NJ (2005). Artemisinin-based combinations.. Curr Opin Infect Dis.

[6] Epstein JE, Giersing B, Mullen G, Moorthy V, Richie TL (2007). Curr Opin Mol Therap.

[7] Ballou WR, Arevalo-Herrera M, Carucci D, Richie TL, Corradin G (2004). Update on the clinical development of candidate malaria vaccines.. Am J Trop Med Hyg.

[8] Heppner DG, Kester KE, Ockenhouse CF, Tornieporth N, Ofori O (2005). Towards an RTS,S-based, multi-stage, multi-antigen vaccine against falciparum malaria: Progress at the Walter Reed Army Institute of Research.. Vaccine.

[9] Kester KE, McKinney DA, Tornieporth N, Ockenhouse CF, Heppner DG (2001). Efficacy of recombinant circumsporozoite protein vaccine regimens against experimental *Plasmodium falciparum* malaria.. J Infect Dis.

[10] Stoute JA, Slaoui M, Heppner DG, Momin P, Kester KE (1997). A preliminary evaluation of a recombinant circumsporozoite protein vaccine against *Plasmodium falciparum* malaria.. N Engl J Med.

[11] Kester KE, McKinney DA, Tornieporth N, Ockenhouse CF, Heppner DG (2007). A phase I/IIa safety, immunogenicity, and efficacy bridging randomized study of a two-dose regimen of liquid and lyophilized formulations of the candidate malaria vaccine RTS,S/AS02_A_ in malaria-naïve adults.. Vaccine.

[12] Kester KE, Cummings JF, Ockenhouse CF, Nielsen R, Hall BT (2008). Phase 2a trial of 0, 1, and 3 month and 0, 7, and 28 day immunization schedules of malaria vaccine RTS,S/AS02 in malaria-naïve adults at the Walter Reed Army Institute of Research.. Vaccine.

[13] Kester KE, Cummings JF, Ofori-Anyiam O, Krzych U, Schwenk R (2009). Randomized, double-blind, Phase 2 trial of falciparum malaria vaccines RTS,S/AS01 and RTS,S/AS02 in malaria-naïve adults; safety, efficacy and immunologic associates of protection.. J Infect Dis.

[14] Stewart VA, McGrath SM, Walsh DS, Davis S, Hess AS (2006). Pre-clinical evaluation of new adjuvant formulations to improve the immunogenicity of the malaria vaccine RTS,S/AS02_A_.. Vaccine.

[15] Stewart VA, Walsh DS, McGrath SM, Kester KE, Cummings JF (2006). Cutaneous delayed-type hypersensitivity (DTH) in a multi-formulation comparator trial of the anti-falciparum malaria vaccine candidate RTS,S in rhesus macaques.. Vaccine.

[16] Mettens P, Dubois PM, Demoitié MA, Bayat B, Donner MN (2007). Improved T cell responses to *Plasmodium falciparum* circumsporozoite protein in mice and monkeys induced by a novel formulation of RTS,S vaccine antigen.. Vaccine.

[17] Polhemus ME, Remich SA, Ogutu BR, Waitumbi JN, Otieno L (2009). Evaluation of RTS,S/AS02_A_ and RTS,S/AS01_B_ in Adults in a High Malaria Transmission Area.. PLoS One.

[18] Alonso PL, Sacarlal J, Aponte JJ, Leach A, Macete E (2004). Efficacy of the RTS,S/AS02_A_ vaccine against *Plasmodium falciparum* infection and disease in young African children: Randomised controlled trial.. Lancet.

[19] Alonso PL, Sacarlal J, Aponte JJ, Leach A, Macete E (2005). Duration of protection with RTS,S/AS02_A_ malaria vaccine in prevention of *Plasmodium falciparum* disease in Mozambican children: Single-blind extended follow-up of a randomised controlled trial.. Lancet.

[20] Aponte JJ, Aide P, Renom M, Mandomando I, Bassat Q (2007). Safety of the RTS,S/AS02D candidate malaria vaccine in infants living in a highly endemic area of Mozambique: a double blind randomised controlled phase I/IIb trial.. Lancet.

[21] Sacarlal J, Aide P, Aponte JA, Renom M, Leach A (2009). Long-term safety and efficacy of the RTS,S/AS02A malaria vaccine in Mozambican children.. J Inf Dis.

[22] Abdulla S, Oberholzer R, Juma O, Kubhoja S, Machera F (2008). Safety and immunogenicity of RTS,S/AS02D Malaria Vaccine in Infants.. N Engl J Med.

[23] Bejon P, Lusingu J, Olotu A, Leach A, Lievens M (2008). Efficacy of RTS,S/AS01E vaccine against malaria in children 5 to 17 months of age.. N Engl J Med.

[24] Escalante AA, Grebert HM, Isea R, Goldman IF, Basco L (2002). A study of genetic diversity in the gene encoding the circumsporozoite protein (CSP) of *Plasmodium falciparum* from different transmission areas—XVI. Asembo Bay Cohort Project.. Mol Biochem Parasitol.

[25] Alloueche A, Milligan P, Conway DJ, Pinder M, Bojang K (2003). Protective efficacy of the RTS,S/AS02 *Plasmodium falciparum* malaria vaccine is not strain specific.. Am J Trop Med Hyg.

[26] Enosse S, Dobano C, Quelhas D, Aponte JJ, Lievens M (2006). RTS,S/AS02_A_ malaria vaccine does not induce parasite CSP T cell epitope selection and reduces multiplicity of infection.. PLoS Clinical Trials.

[27] Snounou G, Zhu X, Siripoon N, Jarra W, Thaithong S (1999). Biased distribution of msp1 and msp2 allelic variants in *Plasmodium falciparum* populations in Thailand.. Transactions of the Royal Society of Tropical Medicine and Hygiene.

[28] Ord R, Alexander N, Dunyo S, Hallett RL, Jawara M (2007). Seasonal carriage of *pfcrt* and *pfmdr1* alleles in Gambian *Plasmodium falciparum* imply reduced fitness of chloroquine-resistant parasites.. J Infect Dis.

[29] Doolan DL, Saul AJ, Good MF (1992). Geographically restricted heterogeneity of the *Plasmodium falciparum* circumsporozoite protein: relevance for vaccine development.. Infect Immun.

[30] Takala SL, Coulibaly D, Thera MA, Dicko A, Smith DL (2007). Dynamics of polymorphism in a malaria vaccine antigen at a vaccine-testing site in Mali.. PLoS Med.

[31] Ofosu-Okyere A, Mackinnon MJ, Sowa MP, Koram KA, Nkrumah F (2001). Novel *Plasmodium falciparum* clones and rising clone multiplicities are associated with the increase in malaria morbidity in Ghanaian children during the transition into the high transmission season.. Parasitology.

[32] Mayor A, Saute F, Aponte JJ, Almeda J, Gomez-Olive FX (2003). *Plasmodium falciparum* multiple infections in Mozambique, its relation to other malariological indices and to prospective risk of malaria morbidity.. Trop Med Int Health.

[33] Farnert A, Rooth I, Svensson A, Snounou G, Bjorkman A (1999). Complexity of *Plasmodium falciparum* infections is consistent over time and protects against clinical disease in Tanzanian children.. J Infect Dis.

[34] Cortes A, Mellombo M, Benet A, Lorry K, Rare L (2004). *Plasmodium falciparum*: Distribution of msp2 genotypes among symptomatic and asymptomatic individuals from the Wosera region of Papua New Guinea.. Exp Parasitol.

