# Supplementary material for: Impact of RTS,S/AS02A and RTS,S/AS01B on Genotypes of P. falciparum in Adults Participating in a Malaria Vaccine Clinical Trial
Source: PLoS One. 2009 Nov 17;4(11):e7849. doi: 10.1371/journal.pone.0007849 (PMC2773849; doi:10.1371/journal.pone.0007849)
Supplement: Figure S1 — Clustalalignment of CSP amino acid haplotypes. (0.22 MB PDF) [file pone.0007849.s001.pdf]

Supplementary Figure S1: Clustal alignment of CSP amino acid haplotypes.

Deduced protein sequence of sequenced region including Th2R and Th3R epitopes, showing 29 different sequences observed among 81 ADI samples analysed, compared to 3D7 (vaccine antigen) sequence. Majority residues are white, minority substitutions are black.

```
3D7CSP    NANANSAVKNNNNEEPSDKHIKEYLNKIQNSLSTEWSPCSVTCGNGIQVRIKPGSANKPKDELDYANDIEKKICKMEKCSSV
0001      NANANNAEKNNNNEEPSDQHIEKYLKIIQNSLSTEWSPCSVTCGNGIQVRIKPGSANKPKDQLDYANDIEKKICKMEKCSSV
0002      NANANNAVKNNNNEEPSDQHIEKYLKIIQNSLSTEWSPCSVTCGNGIQVRIKPGSANKPKDQLDYANDIEKKICKMEKCSSV
0003      NANANNAVKNNNNEEPSDQHIEKYLQKIQNSLSTEWSPCSVTCGNGIQVRIKPGSANKPKDQLNYENDIEKKICKMEKCSSV
0004      NANANNAVKNNNNEEPSDQHIEQYLNRIQNSLSTEWSPCSVTCGNGIQVRIKPGSANKPKDQLNYENDIEKKICKMEKCSSV
0005      NAKANNAVKNNNNEEPSDKHIEQYLKTIQNSLSTEWSPCSVTCGNGIQVRIKPGSANKPKDQLDYENDIEKKICKMEKCSSV
0006      NANANNAVKNNNNEEPSDKHIEQYLKTIQNSLSTEWSPCSVTCGNGIQVRIKPGSANKPKDQLDYENDIEKKICKMEKCSSV
0007      NANANNAVKNNNNEEPSDQHIEQYLKTIQNSLSTEWSPCSVTCGNGIQVRIKPGSANKPKDQLDYENDIEKKICKMEKCSSV
0008      NANANNAVKNNNNEEPSDKHIEEYLKKIKNSISTEWSPCSVTCGNGIQVRIKPGSANKPKDELNYENDIEKKICKMEKCSSV
0009      NANANNAVKNNNNEEPSDKHIEQYLKKIKNSISTEWSPCSVTCGNGIQVRIKPGSANKPKDELDYENDIEKKICKMEKCSSV
0010      NANANNAVKNNNNEEPSDKHIEQYLKKIQNSLSTEWSPCSVTCGNGIQVRIKPGSAGKSKDELDYENDIEKKICKMEKCSSV
0011      NANANNAVKNNNNEEPSDKHIEKYLKEIQNSLSTEWSPCSVTCGNGIQVRIKPGSAGKPKNELDYENDIEKKICKMEKCSSV
0012      NANANNAVKNNNNEEPSDQHIEKYLKKIQNSLSTEWSPCSVTCGNGIQVRIKPGSANKPKDQLDYANDIEKKICKMEKCSSV
0014      NANANNAVKNNNNEEPSDQHIEKYLKKIQNSLSTEWSPCSVTCGNGIQVRIKPGSANKPKDQLDYENDIEKKICKMEKCSSV
0015      NANANNAVKNNNNEEPSDKHIKEYLKIIQNSLSTEWSPCSVTCGNGIQVRIKPGSAGKPKEELNYENDIEKKICKMEKCSSV
0016      NANANNAVKNNNNEEPSDKHIEQYLKTIKNSLSTEWSPCSVTCGNGIQVRIKPGSANKPKDQLDYENDIEKKICKMEKCSSV
0017      NANANNAVKNNNNEEPSDQHIEQYLKTIKNSLSTEWSPCSVTCGNGIQVRIKPGSANKPKDQLDYANDIEKKICKMEKCSSV
0018      NANANNAVKNNNNEEPSDQHIEKYLKIIQNSLSTEWSPCSVTCGNGIQVRIKPGSANKPKDQLDYANDIEKKICKMEKCSSV
0019      NANANNAVKNNNNEEPSDQHIEKYLQKIQNSLSTEWSPCSVTCGNGIQVRIKPGSANKPKDQLDYENDIEKKICKMEKCSSV
0020      NANANNAVKNNNNEEPSDQHIEKYLNKIKNSLSTEWSPCSVTCGNGIQVRIKPGSANKPKDQLDYANDIEKKICKMEKCSSV
0021      NANANNAVKNNNNEEPSDQHIEKYLKIIQNSLSTEWSPCSVTCGNGIQVRIKPGSANKPKDQLDYANDIEKKICKMEKCSSV
0022      NANANNAVKNNNNEEPSDKHIEQYLKKIQNSLSTEWSPCSVTCGNGIQVRIKPGSANKPKDELDYENDIEKKICKMEKCSSV
0023      NANANNAVKNNNNEEPSDKHIEQYLKKIKNSISTEWSPCSVTCGNGIQVRIKPGSANKPKDELDYENDIEKKICKMEKCSSV
0024      NANANNAVKNNNNEEPSDKHIEQYLKKIQNSLSTEWSPCSVTCGNGIQVRIKPGSAGKSKDELDYENDIEKKICKMEKCSSV
0025      NANANNAVKNNNNEEPSDKHIEEYLKKIKNSISTEWSPCSVTCGNGIQVRIKPGSANKPKDELNYENDIEKKICKMEKCSSV
0026      NANANNAVKNNNNEEPSDQHIEKYLQKIQNSLSTEWSPCSVTCGNGIQVRIKPGSANKPKDQLNYENDIEKKICKMEKCSSV
0027      NANANNAVKNNNNEEPSDQHIEQYLKRIQNSLSTEWSPCSVTCGNGIQVRIKPGSANKPKDQLDYANDIEKKICKMEKCSSV
0028      NAKANNAVKNNNNEEPSDKHIEQYLKTIQNSLSTEWSPCSVTCGNGIQVRIKPGSANKPKDQLDYENDIEKKICKMEKCSSV
0029      NANANNAVKNNNNEEPSDKHIKEYLKIIQNSLSTEWSPCSVTCGNGIQVRIKPGSAGKPKEELNYENDIEKKICKMEKCSSV
0030      NANANNAVKNNNNEEPSDKHIEKYLKEIQNSLSTEWSPCSVTCGNGIQVRIKPGSAGKPKNELDYENDIEKKICKMEKCSSV
```

Th2R **Th3R**
