# Supplementary material for: Impact of RTS,S/AS02A and RTS,S/AS01B on Genotypes of P. falciparum in Adults Participating in a Malaria Vaccine Clinical Trial
Source: PLoS One. 2009 Nov 17;4(11):e7849. doi: 10.1371/journal.pone.0007849 (PMC2773849; doi:10.1371/journal.pone.0007849)
Supplement: Table S1 — CSP 3D7/non 3D7 type by polymorphic amino acid site in Th2r (single+majority) (ATP cohort for efficacy) (0.02 MB PDF) [file pone.0007849.s002.pdf]

Supplementary Table S1. CSP 3D7/non 3D7 type by polymorphic amino acid site in Th2r (single+majority) (ATP cohort for efficacy)

		Gr 0 N = 38		Gr 1 N = 15		Gr 2 N = 23		Gr 3 N = 31		P-value (Fisher)		
Characteristics	Parameters or Categories	Value or n	%	Value or n	%	Value or n	%	Value or n	%	Pooled-Control	AS01B - Control	AS02A – Control
K329	Not 3D7	16	42.1	8	53.3	8	34.8	14	45.2			
	3D7	22	57.9	7	46.7	15	65.2	17	54.8	0.8123	0.7549	0.5772
K332	Not 3D7	35	92.1	15	100	20	87.0	27	87.1			
	3D7	3	7.9	0	0.0	3	13.0	4	12.9	0.6925	0.2876	1
E333	Not 3D7	32	84.2	15	100	17	73.9	24	77.4			
	3D7	6	15.8	0	0.0	6	26.1	7	22.6	0.5446	0.0782	1
L335	Not 3D7	0	0.0	0	0.0	0	0.0	1	3.2			
	3D7	38	100	15	100	23	100	30	96.8	0.4493	1	1
N336	Not 3D7	34	89.5	15	100	19	82.6	27	87.1			
	3D7	4	10.5	0	0.0	4	17.4	4	12.9	1	0.2876	0.7108
K337	Not 3D7	28	73.7	14	93.3	14	60.9	21	67.7			
	3D7	10	26.3	1	6.7	9	39.1	10	32.3	0.6055	0.0737	0.7739
Q339	Not 3D7	16	42.1	8	53.3	8	34.8	6	19.4			
	3D7	22	57.9	7	46.7	15	65.2	25	80.6	0.0684	0.0377	0.2259
L342	Not 3D7	7	18.4	0	0.0	7	30.4	4	12.9			
	3D7	31	81.6	15	100	16	69.6	27	87.1	0.7428	0.2876	0.1728

Gr.0 = Pooled RTS,S
Gr.1 = RTS,S/$AS01_B$
Gr.2 = RTS,S/$AS02_A$
Gr.3 = Rabies vaccine
N = number of subjects
n = number of subjects in a given category
Value = value of the considered parameter
% = n / Number of subjects with available results x 100
