# Supplementary material for: Impact of RTS,S/AS02A and RTS,S/AS01B on Genotypes of P. falciparum in Adults Participating in a Malaria Vaccine Clinical Trial
Source: PLoS One. 2009 Nov 17;4(11):e7849. doi: 10.1371/journal.pone.0007849 (PMC2773849; doi:10.1371/journal.pone.0007849)
Supplement: Table S2 — CSP 3D7/non 3D7 type by polymorphic amino acid site in Th3r (single+majority) (ATP cohort for efficacy) (0.02 MB PDF) [file pone.0007849.s003.pdf]

Supplementary Table S2. CSP 3D7/non 3D7 type by polymorphic amino acid site in Th3r (single+majority) (ATP cohort for efficacy)

		Gr 0 N = 38		Gr 1 N = 15		Gr 2 N = 23		Gr 3 N = 31		P-value (Fisher)		
Characteristics	Parameters or Categories	Value or n	%	Value or n	%	Value or n	%	Value or n	%	Pooled-Control	AS01B - Control	AS02A – Control
N367	Not 3D7	6	15.8	1	6.7	5	21.7	11	35.5			
	3D7	32	84.2	14	93.3	18	78.3	20	64.5	0.0912	0.0702	0.3701
P369	Not 3D7	1	2.6	0	0.0	1	4.3	3	9.7			
	3D7	37	97.4	15	100	22	95.7	28	90.3	0.3194	0.5405	0.6280
D371	Not 3D7	1	2.6	0	0.0	1	4.3	7	22.6			
	3D7	37	97.4	15	100	22	95.7	24	77.4	0.0187	0.0782	0.1186
E372	Not 3D7	29	76.3	13	86.7	16	69.6	21	67.7			
	3D7	9	23.7	2	13.3	7	30.4	10	32.3	0.5887	0.2846	1
D374	Not 3D7	4	10.5	1	6.7	3	13.0	3	9.7			
	3D7	34	89.5	14	93.3	20	87.0	28	90.3	1	1	1
A376	Not 3D7	21	55.3	7	46.7	14	60.9	20	64.5			
	3D7	17	44.7	8	53.3	9	39.1	11	35.5	0.4699	0.3412	1

Gr.0 = Pooled RTS,S
Gr.1 = RTS,S/AS01$_B$
Gr.2 = RTS,S/AS02$_A$
Gr.3 = Rabies vaccine
N = number of subjects
n = number of subjects in a given category
Value = value of the considered parameter
% = n / Number of subjects with available results x 100
