# Supplementary material for: Impact of RTS,S/AS02A and RTS,S/AS01B on Genotypes of P. falciparum in Adults Participating in a Malaria Vaccine Clinical Trial
Source: PLoS One. 2009 Nov 17;4(11):e7849. doi: 10.1371/journal.pone.0007849 (PMC2773849; doi:10.1371/journal.pone.0007849)
Supplement: Table S3 — Th2R and Th3R haplotype prevalence at baseline and among ADI samples. (0.08 MB PDF) [file pone.0007849.s004.pdf]

Th2R haplotypes

Pre-vaccination July 05											ADI Oct 05-Dec 06		
Th2	Freq	Percent	K329	K332	E333	L335	N336	K337	Q339	L342	Th2	Freq.	Percent
00000111	0	0									00000111	1	1.23
00010001	7	4.79									00010001	5	6.17
00010011	22	15.07									00010011	20	24.69
00010111	6	4.11									00010111	6	7.41
00011101	6	4.11									00011101	1	1.23
10010001	15	10.27									10010001	12	14.81
10010011	25	17.12									10010011	14	17.28
10010100	4	2.74									10010100	3	3.7
10010111	49	33.56									10010111	0	0
10011100	1	0.68									10011100	3	3.7
10110011	2	1.37									10110011	3	3.7
10110100	4	2.74									10110100	2	2.47
10110111	0	0									10110111	2	2.47
11110011	2	1.37									11110011	2	2.47
11111110	2	1.37									11111110	7	8.64
11111111	1	0.68									11111111	0	0
Total	146	100									Total	81	100

Th3R haplotypes

Pre-vaccination July 05									ADI Oct 05-Dec 06		
Th3	Freq.	Percent	N367	P369	D371	E372	D374	A376	Th3	Freq.	Percent
000110	2	1.37							000110	5	6.17
001110	1	0.68							001110	0	0
010100	2	1.37							010100	1	1.23
010110	2	1.37							010110	4	4.94
011010	0	0							011010	4	4.94
011011	5	3.42							011011	5	6.17
011111	2	1.37							011111	1	1.23
111000	5	3.42							111000	2	2.47
111010	29	19.86							111010	23	28.4
111011	37	25.34							111011	24	29.63
111100	4	2.74							111100	6	7.41
111110	55	37.67							111110	5	6.17
111111	2	1.37							111111	1	1.23
Total	146	100							Total	81	100

Supplementary Table 3: Th2R and Th3R haplotype prevalence at baseline and among ADI samples.
Binary codes for each haplotype are given. in vertical columns; polymorphic codons in each epitope are set out along the top of each table. Shaded cells indicate that the 3D7 allele is present at that position.
